# Complementary Roles of the Hippocampus and the Dorsomedial Striatum during Spatial and Sequence-Based Navigation Behavior

**DOI:** 10.1371/journal.pone.0067232

**Published:** 2013-06-27

**Authors:** Céline Fouquet, Bénédicte M. Babayan, Aurélie Watilliaux, Bruno Bontempi, Christine Tobin, Laure Rondi-Reig

**Affiliations:** 1 CNRS-Université Pierre et Marie Curie-P6, ENMVI team, UMR7102, Paris, France; 2 CNRS-Université Victor Segalen – Bordeaux 2, Institut des Maladies neurodégénératives, UMR5293, Bordeaux, France; Duke University, United States of America

## Abstract

We investigated the neural bases of navigation based on spatial or sequential egocentric representation during the completion of the starmaze, a complex goal-directed navigation task. In this maze, mice had to swim along a path composed of three choice points to find a hidden platform. As reported previously, this task can be solved by using two hippocampal-dependent strategies encoded in parallel i) the allocentric strategy requiring encoding of the contextual information, and ii) the sequential egocentric strategy requiring temporal encoding of a sequence of successive body movements associated to specific choice points. Mice were trained during one day and tested the following day in a single probe trial to reveal which of the two strategies was spontaneously preferred by each animal. Imaging of the activity-dependent gene c-fos revealed that both strategies are supported by an overlapping network involving the dorsal hippocampus, the dorsomedial striatum (DMS) and the medial prefrontal cortex. A significant higher activation of the ventral CA1 subregion was observed when mice used the sequential egocentric strategy. To investigate the potential different roles of the dorsal hippocampus and the DMS in both types of navigation, we performed region-specific excitotoxic lesions of each of these two structures. Dorsal hippocampus lesioned mice were unable to optimally learn the sequence but improved their performances by developing a serial strategy instead. DMS lesioned mice were severely impaired, failing to learn the task. Our data support the view that the hippocampus organizes information into a spatio-temporal representation, which can then be used by the DMS to perform goal-directed navigation.

## Introduction

Extensive research in rodents using navigation tasks has made a distinction between simple egocentric (response learning based on stimulus response-like associations) and allocentric (or place-learning) navigation [1]. In such tasks, the dorso-striatal system mediates habit or response learning [2], [3], [4] and the hippocampal system predominantly supports place learning [5], [6].

This parallel functioning of the dorsal striatum and the hippocampus is however not always clear-cut as revealed by recent findings. On one hand, the dorsal striatum has been shown to be anatomically [7] and functionally divided in a dorsolateral and a dorsomedial part, the former being related to response learning and the latter mediating goal-directed learning [8]. On the other hand, using a more complex paradigm, the starmaze task, we previously demonstrated that an additional strategy we called *sequential egocentric* is encoded concomitantly with the allocentric strategy [9], [10]. Sequential egocentric strategy differs from a simple egocentric strategy as it requires a temporal order memory of successive choice points [11]. Due to its sequential nature, this strategy depends on the hippocampus in both rodent [9] and human [12]. In this study, we hypothesized that the hippocampus and the dorsomedial striatum (DMS) could support together a spatial task which can be solved by mice using either an allocentric or a sequential egocentric strategy.

One fundamental characteristic of hippocampal function is to enable the relational organization of declarative memory [13], [14], [15], explaining its involvement in the sequential egocentric strategy. In rodents, place cell properties provide physiological correlates of such relational organization of the spatio-temporal representation in the hippocampus. For example, the experience of moving from one location to another along a route can be represented by the resulting sequence of place fields traversed [16]. Moreover, there has been a growing body of evidence that the hippocampus is essential to memorize sequences of events [17], [18], [19], [20], [21], [22]. Likewise in humans, functional magnetic resonance imaging (fMRI) studies provided converging evidence that retrieving the temporal order of a series of events recruits preferentially the hippocampus [23], [24], [25], [26], [12].

Concerning the DMS, the first demonstration of its implication in goal-directed learning comes from instrumental conditioning [27], see review in [28]. In a spatial task, actions need to be organized to reach a specific goal as well, thus questioning the possible role of DMS in spatial learning. Indeed rats with a lesion of the DMS were found to be impaired in a water maze [29] and Yin and Knowlton [30] nuanced Packard and McGaugh experiment [2] by showing that the DMS is needed to solve the simple cross-maze experiment by using place learning. Using a spatial alternation task, Moussa et al. [31] proposed that the DMS is necessary to link the goal with the suitable spatial behavior. From a computational point of view, this has been formalized as a role of the DMS in navigation strategies based on an internal model of the full sequence of movements to reach the goal, no matter if such internal model is allocentric or egocentric [32].

Functional connectivity between the DMS and the hippocampus was demonstrated in rodents during the choice period in a T-maze task [33] and in human for successful contextually dependent navigation [34]. Interaction between the human hippocampus and the caudate nucleus (homologous to the rodent DMS) has also been shown during route recognition [35]. No direct anatomical connectivity is known between the DMS and the hippocampal formation however these structures have been shown to interact through the prefrontal cortex on the one hand [36], [37], [38] and through the ventral striatum and substantia nigra (pars compacta) on the other hand [39] (see review in [7] and [40]). Gruber and McDonald [41] suggested that the DMS is an important node for translating hippocampal information into optimal goal-directed navigation.

We first examined the possible co-activation of the DMS with the hippocampus in the allocentric as well as the sequential egocentric strategies in the starmaze task. The use of either strategy can be detected accurately by analyzing the response made in a subsequent probe trial for which the start point is changed [9]. Thus mice can reach the hidden platform by remembering either its spatial position using the allocentric strategy, or the sequence of successive choice points leading to the goal, using the sequential egocentric strategy. To map the neuronal activity of the hippocampal and DMS memory systems, we used cellular imaging of the activity-dependent gene c-fos [42], [43]. We then adopted an invasive approach by performing region-specific excitotoxic lesions of either the dorsal hippocampus or the DMS in order to determine the effects of these selective lesions on learning the goal-directed navigation task in the starmaze. Our results suggest that the hippocampus organizes information into a spatio-temporal representation, which can then be used by the DMS to link a goal to an optimal path.

## Materials and Methods

### Ethics Statement

All behavioral experiments were performed in accordance with the official European guidelines for the care and use of laboratory animals (86/609/EEC) and in accordance with the Policies of the French Committee of Ethics (Decrees n° 87–848 and n° 2001–464). Animal housing facility of the laboratory is fully accredited by the French Direction of Veterinary Services (n°B-75-05-24, 18/05/2010). Animal surgery and experimentation are authorized by the French Direction of Veterinary Services for LRR (# 75-752, 2009) and for AW (# 75-1634, 2009).

All efforts were made to minimize suffering and animal discomfort. Prior to surgery, mice were anesthetized by intraperitoneal injection of ketamine/xylazine. After surgery, mice were allowed 15-day rest and were observed regularly during this period to check if there was no infection and no weight loss.

At the end of the experiment, mice were sacrificed for Fos imaging and lesion analyses (see *Brain extraction for Fos imaging and histological verification of lesions* section). Mice were deeply anesthetized by intraperitoneal injection of ketamine/xylazine and perfused transcardially to extract the brain.

### Experimental Subjects

Seventy one male C57BL/6J mice (3-month-old; Janvier, France) were used throughout the experiment (n = 24 in the Fos imaging study and n = 47 in the lesion study). Mice were housed in groups of 5 in standard conditions: 12 h light/dark cycle, with *ad libitum* access to water and food. Seven days prior the beginning of sensory-motor tests, mice were separated and housed individually to limit the inter-boxes variability resulting from social relationships. All behavioral experiments took place during the light cycle.

### Behavioral Studies

Prior to training in the starmaze task, motor coordination and balance were evaluated in an accelerating rotarod (see [44]).

**Figure 1:**
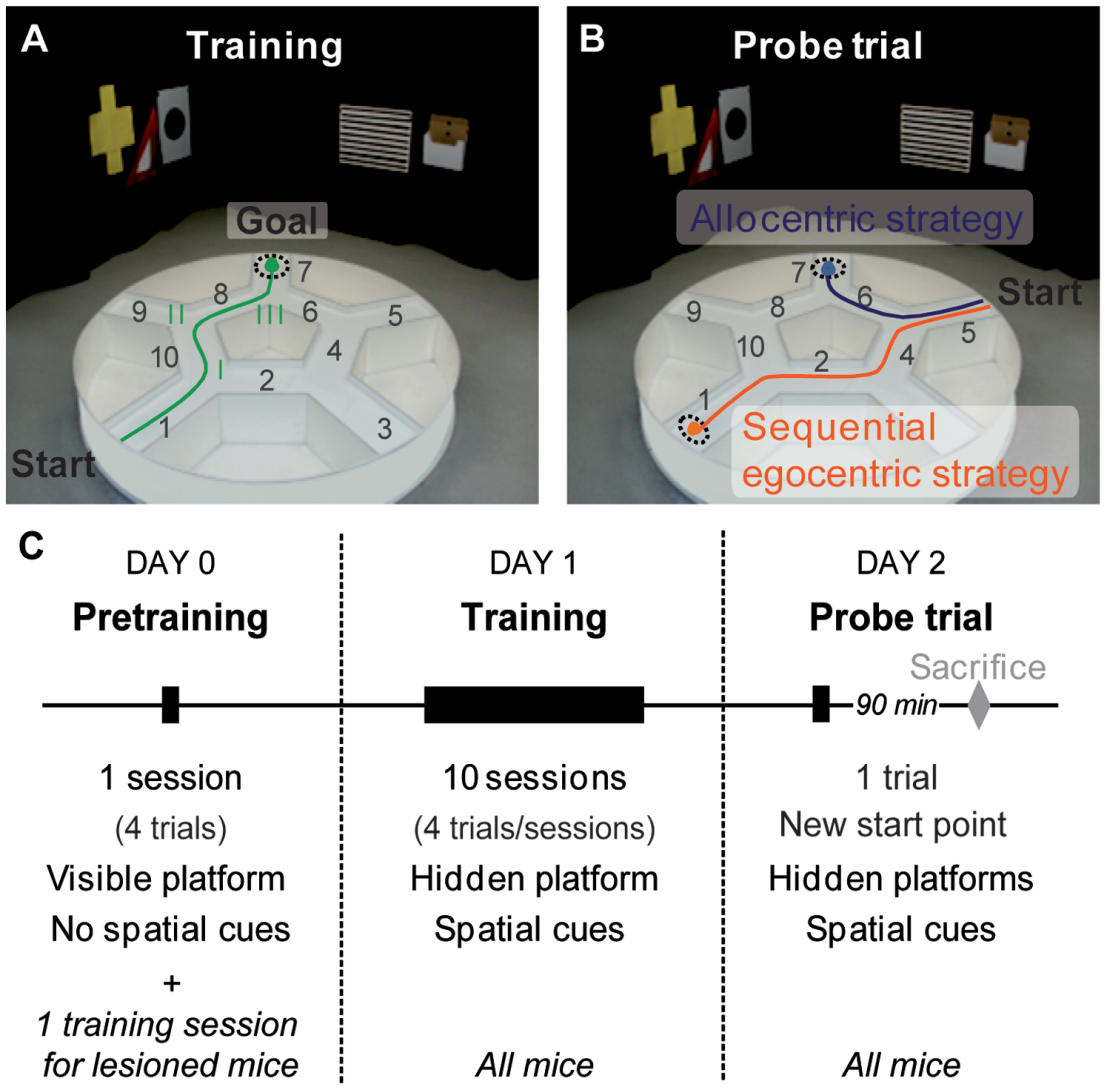
Experimental procedures in the starmaze. (**A**) Mice placed in a fixed departure alley (1) were trained to reach a fixed escape platform (dashed circle in alley 7) submerged under opaque water in the presence of visual spatial cues. The direct path to the platform is drawn in green. Intersections are numbered in roman numerals. (**B**) The probe trial. The day after (Day 2) the training day (Day 1), each mouse underwent a probe trial allowing to identify the strategy spontaneously used during training. The animal was placed in a new departure alley (alley 5) and could reach one of the invisible platforms located either in alley 1 or alley 7 in the presence of visual spatial cues. Mice that reached the habitual goal location (blue line) used an allocentric strategy whereas mice reproducing the temporal sequence of body movements (turning left, right and left; orange line) reaching alley 1 used the sequential egocentric strategy (orange line). (**C**) Design of the behavioral assay. The pretraining phase consists in reaching a visible platform (cued with a flag) from different departure points during a session of 4 trials the day before training, in the absence of any spatial cues (Day 0). The training procedure (Day 1) was composed of 10 sessions of 4-trials with an inter-trial-interval of 10 min and an inter-session interval of 40 min. On day 2, a single probe trial was given prior to processing the brains for Fos immunostaining. For the lesioned mice, a training session (4 trials with a hidden platform and visual spatial cues) is included on Day 0, after the pretraining to insure an optimal learning during the training phase.

The starmaze is a complex goal-directed navigation task which consists of five alleys forming a central pentagonal ring and five alleys radiating from the vertices of this pentagonal ring (see [9] for details). All alleys were filled with water made opaque with an inert nontoxic product (Accuscan OP 301, Brenntage, Lyon, France) (Fig. 1A). White noise (50–60 dB) was used to cover all other sounds that the mice could have used to orientate themselves. The maze was surrounded by a square black curtain with 2-D and 3-D patterns affixed to provide configurations of spatial cues. To solve the starmaze task, mice had to swim to a platform hidden 1 cm below the water surface. The departure (alley 1) and arrival (alley 7) points were fixed (Fig. 1A). When a mouse reached the platform, it was allowed to spend 30 sec on it. If an animal did not locate the escape platform within the maximum swimming time (60 sec), the experimenter placed the animal on the platform where it remained for 30 sec.

#### The starmaze task: training schedule (Fig. 1C)

The starmaze task relied on a massed training phase given over one day (day 1), composed of 10 sessions of 4 trials in the presence of all visual cues (see Fig. 1C for the training schedule).

The day before the training phase (day 0), all mice were given a pretraining session of 4 trials in the starmaze to limit stress and habituate them to swim and climb on the platform. The pretraining session was a cued version of the starmaze in which a proximal cue (a flag) was placed on the top of the submerged platform in the absence of any other visual cues (the starmaze was surrounded by a circular black curtain). On day 0, an additional training session was run on all mice included in the lesion study to insure optimal learning during the training phase.

#### Probe trial (Fig. 1B)

The day following training (day 2), a probe trial was run to identify the strategy spontaneously used by each animal (Fig. 1B), namely the allocentric or the sequential egocentric strategy. To be interpretable the probe trial requires a perfect acquisition of the task. Thus, only the probe trials of mice which had reached the learning criterion were analyzed. This criterion was defined as no more than 1 error (i.e. 1 wrong turn) during the last 4 trials on day 1.

The departure point was changed (alley 5). In addition to the habitual platform located in alley 7, another hidden platform was placed in alley 1 so that both strategies were equally rewarded. If a mouse used the configuration of distal visual cues, it performed the [5–6–7] trajectory revealing the use of an allocentric strategy. Executing the temporal sequence of body movements (left–right–left) led to the [5–4–2–1] trajectory indicating a sequential egocentric strategy. Any other path was classified as 'other strategy'.

#### Analysis of learning: direct path score and behavioral strategies used

To evaluate the mouse’s ability to learn the correct sequence of turns and thus to elaborate a direct path towards the platform (Fig. 1A), we computed the direct path score relying on the turn scores for the intersection I, II and III. The turn score reflects the ability of making the correct turn at the first encounter with a given intersection only when following the ideal path (i.e. when coming from alley 1 for intersection I, alley 10 for intersection II, alley 8 for intersection III; Fig. 1A). If the mouse makes a correct turn, it is given a score of 100. A score of 0 is given otherwise. To calculate the direct path score, each turn score is normalized by the number of alleys visited between the evaluated intersection and the first encounter with the previous one. The normalized scores for the three intersections are then averaged. This enables to evaluate the ability of the mouse to navigate directly from one intersection to another. This score thus takes into account the ability of doing the correct turns, but also more generally the ability of mice to orientate themselves towards the platform, especially at the first intersection. Indeed the configuration of the starmaze is such that when a mouse turns right at the first intersection, it goes towards the opposite direction from the location of the platform. The direct path score ranges from 0 to 100 and equals 100 when the path is direct (alleys 1-10-8-7) [45]. Additional examples are given in table S1.

The mice were classified according to their most used path during the last five training sessions, i.e. the second part of training. Different categories were used: “Direct path” corresponding to the most direct path to the platform (alleys 1-10-8-7, see Fig. 1A, green arrow) which can be achieved using either optimal strategy, allocentric or sequential egocentric. This analysis revealed additional non optimal ways of performing the task: “Long path” corresponding to a path along the alleys 1-2-4-6-7, “Serial strategy” consisting in visiting all radiating alleys successively until finding the platform (alleys 1-10-9-8-7), and “No strategy”, when mice did not repeat any clear path.

### Control Animals Used for the Fos Imaging Study

In order to evaluate Fos expression specifically related to task learning, free swimming control mice were employed. These control mice were placed in the departure alley (31×25 cm) blocked with Plexiglas walls in presence of all visual cues (Fig. S1) and allowed to swim for the averaged escape latency of the experimental group. No escape platform was present. As Fos expression is known to be sensitive to environmental novelty [46], placing the control animals in the same new alley as the experimental mice for the probe trial, i.e. alley 5 (Fig. 1B and Fig. S1), allowed to take into consideration Fos expression induced by the change of view point and novelty effect. Thus, these animals allowed to estimate Fos expression related to the non mnemonic aspects of the testing procedure such as the change of view point, stress and motor behavior. Contrasting the mice using the allocentric or sequential egocentric strategy to the control group allowed us to identify structures involved in both memory reactivation and use of a strategy.

### Surgery

Mice were randomly assigned to one of four groups: hippocampus sham [Hipp PBS] (n = 15), hippocampus lesion [Hipp IBO] (n = 14), dorsomedial striatum sham [DMS PBS] (n = 10), dorsomedial striatum lesion [DMS IBO] (n = 8). Excitotoxic lesions were performed by means of bilateral microinjections of ibotenic acid (Sigma, St. Louis, MO, USA) dissolved in phosphate-buffered saline (pH 7.4; 10 g/l) and sham lesions consisted in PBS 1X (Phosphate Buffer Saline) solution injections. Mice were anesthetized with ketamine (Imalgène 1000, Merial, Lyon, France; 100 mg/kg i.p.) and xylazine (Rompun, Bayer, KVP Kiel, Germany; 10 mg/kg i.p.) and placed in a stereotaxic frame (Stoelting). The skull was exposed and holes were drilled above injection sites. Targeting coordinates were determined from the atlas of Franklin and Paxinos [47]. Coordinates were measured in relation to bregma and the skull surface. For the hippocampus, 0.3 µL was injected per side (Antero-Posterior [AP],−2 mm; Medio-Lateral [ML], +/−1.4 mm; Dorso-Ventral [DV], −1.8 mm). For the dorsomedial striatum, 0.48 µL was injected per side (AP, +0.75 mm; ML, +/−1.3 mm; DV, −3.0 mm). Injections (80 nL/min) were made through a glass capillary 1 min after lowering it into the target region using a hydraulic micromanipulator (Narishige, Japan). After each injection, the capillary was left in place for another minute. After completing the injections, the scalp was sutured, and the mouse returned to its home cage for two weeks of resting. The mice were then handled daily (approximately 5 min/day) for a week and tested in the rotarod task at the end of the week. Starmaze testing began three weeks after surgery.

### Brain Extraction for Fos Imaging and Histological Verification of Lesions

Ninety minutes after the completion of the last probe trial (or free swimming session) in the starmaze, mice were deeply anesthetized with an intraperitoneal injection of a mixture of Ketamine (150 mg/kg) and Xylazine (12 mg/kg) and perfused transcardially with saline (0.9%), followed by an ice-cold solution of 4% paraformaldehyde in phosphate buffer (PB 0.1 M, pH 7.4). After postfixation overnight in the same fixative at 4°C, brains were cryoprotected for 48 hours in a sucrose solution (30% in PB 0.1 M, pH7.4) at 4°C. 50 µm-thick coronal sections were cut on a freezing microtome and stored in PB 0.1 M solution containing 0.02% of sodium azide.

### Fos Imaging

Free floating sections were rinsed in 0.1 M PB and then incubated for 30 min with H_2_O_2_ hydrogen peroxide (0.3% in PB). After four 10 min PB-rinses, sections were incubated overnight with a Fos-specific primary rabbit polyclonal antibody (1∶5000, Santa Cruz) diluted in blocking solution (PB 0.1 M, 0.1% BSA, goat serum 2%, 0.2% Triton X-100). A biotinylated goat anti-rabbit antibody (1∶2000, Jackson Immunoresearch) was used as secondary. After washing, staining was revealed using the avidin-biotin peroxidase method (ABC kit, Vectastain Elite kit, Vector Laboratories, Burlingame, CA, USA) coupled to diaminobenzidine as chromogen. Sections were mounted on gelatin-coated slides. Quantitative analyses of Fos-positive nuclei were performed by using a computerized image processing system (Mercator, Exploranova, La Rochelle) coupled to an optical microscope. The quantification of Fos positive nuclei was carried out at 10x magnification. Structures were defined according to the Franklin and Paxinos atlas [47]. Immunoreactive neurons were counted bilaterally using a minimum of three sections, spaced 200 µm from each other. The number of Fos-positive nuclei was quantified in the following areas of interest: subfields CA1, CA3 and dentate gyrus (DG) of the dorsal and ventral hippocampus (dCA1, dCA3, dDG, vCA1, vCA3, vDG), dorsomedial (DMS) and dorsolateral (DLS) parts of the striatum, nucleus accumbens shell (AccS) and core (AccC), prelimbic (PL), infralimbic (IL), parietal (Par), lateral entorhinal (LatEnt) and granular and dysgranular retrosplenial cortices (DysRSP and GrRSP, respectively). The mean count in each structure for each animal (number of Fos positive nuclei per mm^2^) was divided by the mean count in that region of the respective control group to generate a normalized count for each animal. Results expressed as a percentage were averaged to give the final means of each group.

### Histological Verification of Lesions

Free floating sections were incubated overnight at room temperature with a mouse primary antibody against neuronal-specific nuclear protein NeuN (anti-NeuN Alexa Fluor 488 conjugated; 1∶1000; Chemicon, Temecula, CA, USA) in blocking solution (PB 0.1 M, 0.1% BSA, goat serum 2%, 0.2% Triton X-100). Sections were mounted on gelatin-coated slides and coverslipped with Vectashield mounting medium (Vector labs, Burlingame, CA, USA). Analyses of lesion extent were performed using a computerized image processing system (Mercator, Exploranova, La Rochelle) coupled to a fluorescence microscope. One mouse injected with ibotenic acid in the dorsal hippocampus and five mice injected with ibotenic acid in the dorsomedial striatum (DMS) were excluded of the study due to absence of lesion.

### Data Analysis

Results were expressed as means ± standard error mean (S.E.M.). Statistical analyses were run using the Statview 5.0 software. Learning performances were compared using analyses of variance with repeated measures (two-way repeated ANOVA and one-way ANOVA). Differences in normalized Fos density were assessed using Kruskal-Wallis non parametric tests followed by Mann-Whitney comparisons when indicated (values of p<0.05 were considered as significant).

## Results

### Fos Imaging Study

#### Identification of the navigational strategies used in the starmaze

**Figure 2:**
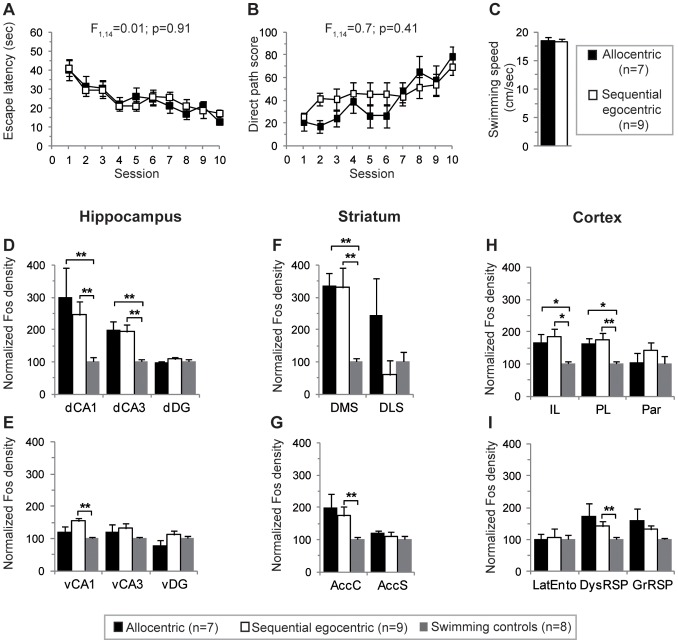
Identification of the network supporting allocentric and sequential egocentric strategies. Mice were categorized as allocentric (n = 7) or sequential egocentric (n = 9) according to their pattern of search in a probe test given one day following training in the starmaze. (**A**) The escape latency to reach the hidden platform declined similarly across sessions in allocentric (closed squares) and sequential egocentric (open squares) mice. (**B**) Their learning profiles were similar as shown by direct path score across learning. (**C**) There was no difference in swimming speed. (**D–I**) Normalized Fos density relative to controls in hippocampal (**D, E**), striatal (**F, G**) and cortical (**H, I**) brain regions of allocentric (black bars, n = 7), sequential egocentric (white bars, n = 9) and swimming control mice (grey bars, n = 8). Note the increased Fos expression in the dorsal CA1 and CA3 fields of the hippocampus, in the dorsomedial striatum (DMS) and the infralimbic (IL) and prelimbic (PL) cortices of the allocentric and the sequential egocentric mice compared to the swimming control group. Compared to control mice, sequential egocentric mice have supplementary increased Fos expression in the ventral CA1 field, the core of the accumbens and the dysgranular retrosplenial cortex. No significant increase in Fos expression was observed in the dentate gyrus (Dorsal DG) and dorsolateral striatum (DLS). Error bars represent s.e.m. * p<0.05, ** p<0.01 Mann Whitney.

Upon completion of training, learning using either the allocentric or the sequential egocentric strategy led to the same direct path (see arrow in Fig. 1A). Each mouse was categorized according to the strategy used during the probe trial performed after the acquisition phase on day 2 (Fig. 1B, C). No significant strategy preference was observed in this experiment as equivalent proportions of mice used allocentric (n = 7 i.e. 44%) or sequential egocentric (n = 9 i.e. 56%) navigational strategies. Their learning profile was similar regardless of the strategy used during navigation as shown by comparable escape latencies (two-way repeated ANOVA, F_1,14_ = 0.01, p = 0.91; Fig. 2A) and direct path score, reflecting the ability of mice to execute the direct path to the platform (two-way repeated ANOVA, *F*_1,14_ = 0.7, *p* = 0.41; Fig. 2B). In addition, no difference in swimming speed was found (unpaired t-test, *t*_14_ = 0.23, *p* = 0.82; Fig. 2C).

#### An overlapping network supports allocentric and sequential egocentric strategies

Fos immunoreactivity was quantified in different structures known to be critical for learning spatial tasks, i.e. the dorsal hippocampus [dCA1, dCA3 and dentate gyrus (dDG) Fig. 2D], the ventral hippocampus [vCA1, vCA3 and dentate gyrus (vDG), Fig. 2E], the dorsal striatum [dorsomedial (DMS) and dorsolateral (DLS) Fig. 2F], the ventral striatum [nucleus accumbens core (AccC) and shell (AccS) Fig. 2G], and different cortices [infralimbic (IL), prelimbic (PL), parietal (Par), lateral entorhinal (LatEnto), dysgranular and granular retrosplenial (GrRSP and DysRSP, respectively) Fig. 2H, I]. To isolate changes in gene expression associated with the non mnemonic aspects of the testing procedure (e.g. locomotor activity, contextual arousal, and change in viewpoint during the probe test), we added a group of control mice which could swim freely in one alley (31×25 cm) of the starmaze without a platform (Fig.S1). Number of Fos-positive nuclei in allocentric (n = 7) and sequential egocentric (n = 9) groups were normalized with respect to the level of Fos expression in control mice (n = 8).

Fos expression analysis in the hippocampus revealed that the dorsal CA1 and CA3 regions were conjointly recruited regardless of the strategy adopted by the mice (Kruskal-Wallis, *p*<0.01, Mann-Whitney *U*, *p*<0.01 for all comparisons to the swimming controls, allocentric versus sequential egocentric Mann-Whitney *U*, *p* = 0.79 for dCA1 and *p* = 0.96 for dCA3; Fig. 2D). However, only sequential egocentric strategy was associated to a significant increase in ventral CA1 (Kruskal-Wallis, *p*<0.01, sequential egocentric versus swimming controls Mann-Whitney *U*, *p*<0.01; Fig. 2E). No significant increase in Fos expression was observed in the DG in either dorsal or ventral subregion as well as in the ventral CA3 compared to the control group (Fig. 2 D, E). When examining Fos expression in the striatum (Fig. 2F), we observed that only the dorsomedial part is recruited, and this in both allocentric and sequential egocentric mice (Kruskal-Wallis, *p*<0.01, Mann-Whitney *U*, *p*<0.01 for all comparisons to the swimming controls for the DMS). We did not observe any Fos activation in the dorsolateral striatum compared to the control group. As for Fos counts in the dorsal CA1 and CA3, Fos counts in the DMS were similar in allocentric and sequential egocentric group (allocentric versus sequential egocentric Mann-Whitney *U*, *p* = 0.63; Fig. 2F). Interestingly, Fos expression in hippocampal areas correlated positively with that in the DMS (Spearman correlation *r* = 0.61, *p*<0.01 for CA1 and *r* = 0.74, *p*<0.01 for CA3; Fig. S2).

There was no clear difference in Fos activation in AccS between controls and mice performing the task while a significant activation was seen in the AccC of mice using the sequential egocentric strategy (Kruskal-Wallis, *p* = 0.04, sequential egocentric versus swimming controls Mann-Whitney *U*, *p*<0.01, allocentric versus swimming controls Mann-Whitney *U*, *p* = 0.1 for AccC; Fig. 2G). Fos activation was significantly higher in IL and PL cortices of mice learning the task compared to controls (Kruskal-Wallis, *p*<0.05, Mann-Whitney *U*, *p*<0.05 for all comparisons to the swimming controls; Fig. 2H). The DysRSP was activated in mice using the sequential egocentric strategy, its recruitment being also close to significance in allocentric mice (Kruskal-Wallis, *p* = 0.04, sequential egocentric versus swimming controls Mann-Whitney *U*, *p* = 0.02, allocentric versus swimming controls Mann-Whitney *U*, *p* = 0.06 for DysRSP; Fig. 2I).

Taken collectively, these results show that allocentric and sequential egocentric strategies in the starmaze were supported by an overlapping structural network which includes the dCA1, dCA3, DMS and medial prefrontal brain regions. Only the vCA1, the accumbens core and the dysgranular retrosplenial cortex were preferentially activated during the sequential egocentric strategy.

### Hippocampal and DMS Lesion Study

The Fos imaging results reported above suggest that the hippocampus and the DMS are part of a common anatomo-functional basis for allocentric and sequential egocentric strategies and raise the possibility that the hippocampus and the DMS play complementary roles during learning of the starmaze goal-directed task. To test this hypothesis, we performed region-specific brain lesions using the excitotoxic agent ibotenic acid prior the starmaze procedure. Motor coordination and balance were evaluated in a rotarod [44]. We did not find any deleterious effect of ibotenic acid injections on motor abilities in either hippocampal or DMS-lesioned mice. The performances of lesioned and sham control mice (injected with PBS) were comparable to those of the mice trained in the Fos imaging study (One-pair ANOVA *F*_5,57_ = 2.23, *p* = 0.06 for time spent on the rod; Fig. S3). The DMS PBS group exhibited the lowest score (60±6 sec on the Rotarod compared to approximately 80 sec in the other groups). However such difference did not affect the starmaze performances of this group (see Fig. 4, B, C and D).

#### Dorsal hippocampus lesion impairs learning in the starmaze task and results in serial strategy learning

**Figure 3:**
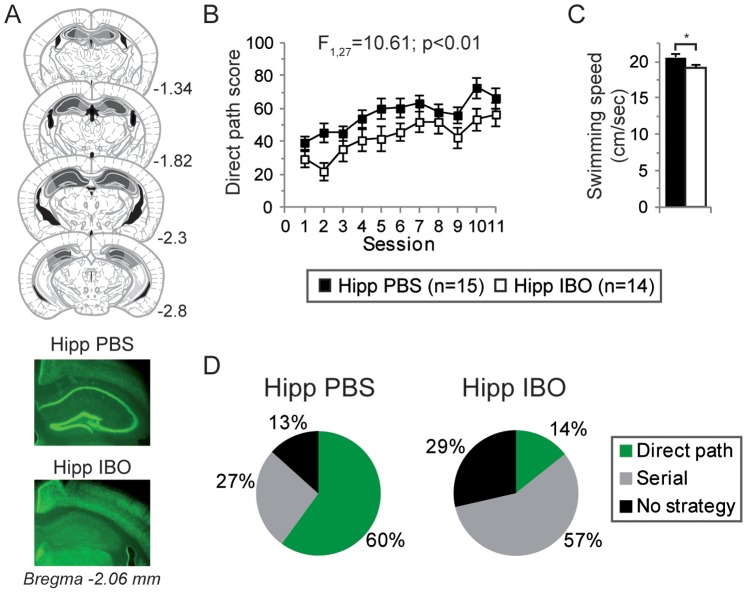
Learning performances of dorsal hippocampal lesioned mice in the starmaze. (**A**) *Upper*. Schematic representation of excitotoxic lesions of the dorsal hippocampus. Shaded areas represent the minimum (dark gray) and the maximum (light gray) extent of the lesions. Ventricles are in black. Numbers indicate distance from Bregma in mm. *Lower.* Representative coronal sections from control (injected with PBS as vehicle, Hipp PBS) and hippocampal mice (injected with ibotenic acid, Hipp IBO) stained for the neuron-specific marker NeuN (mm posterior to bregma). Infusion of ibotenic acid resulted in a near complete neuronal depletion in the CA fields and the dentate gyrus of the dorsal hippocampus. (**B**) Corresponding learning profiles in the starmaze. Compared to PBS-injected mice (closed squares, n = 15), lesioned mice (open squares, n = 14) exhibited an impaired direct path score. (**C**) There was a significant difference in swimming speed (black bar: control mice; white bar: hippocampal mice) but which was not correlated to the direct path score (see Results section). (**D**) Paths most used by the mice during the second half of the training day. Control mice injected with PBS essentially relied on the direct path (60%) whereas 57% of hippocampal mice relied on the serial strategy (turning always left at all intersection) and 29% expressed no clear strategy.

We first targeted the dorsal hippocampus and found that our lesion procedure resulted in a near complete neuronal depletion in the dorsal hippocampus with no detectable damage to surrounding areas (Fig. 3A).

The direct path score differed significantly between hippocampal mice (n = 14) [Hipp IBO] and the sham control mice (n = 15) [Hipp PBS] (two-way repeated ANOVA, *F*_1,27_ = 10.61, *p*<0.01; Fig. 3B). There was a significant difference in swimming speed, the control mice swimming at a higher pace (unpaired t-test, *t*_27_ = -2.39, *p* = 0.02; Fig. 3C). However the swimming speed and the direct path score were not correlated (Spearman correlation *r* = 0.25, *p* = 0.18). Thus, the possibility that the difference in direct path score was due to the difference in swimming speed was unlikely. When analyzing the evolution of the direct path score across sessions, control and lesioned groups both improved their scores during training (one-way repeated ANOVA across sessions for control mice, *F*_10,154_ = 3.37, *p*<0.01; for lesioned mice, *F*_10,143_ = 3.95, *p*<0.01; Fig. 3B).

To further characterize the nature of the deficit of hippocampal mice, we examined how mice behaved during the second part of the training. Two strategies were quantified: the direct path toward the goal or the serial strategy. All other types of behavior were characterized as no clear strategy. There was a significant difference in the distributions of strategies between hippocampal and control mice (Fisher’s exact test, *p* = 0.04). Control mice with PBS injection essentially relied on the direct path (60%). 27% of them used a serial strategy and 13% no clear strategy. By contrast, only two mice (14% of the hippocampal mice) used the direct path. However during the probe test, one of these two mice used a serial strategy while the other did not use any clear strategy. Thus in the hippocampal group, no mice were clearly identified as using the allocentric or the sequential egocentric strategy. 57% of them relied on the serial strategy (visiting all radiating alleys until finding the platform) and 29% even expressed no clear strategy (Fig. 3D).

**Figure 4:**
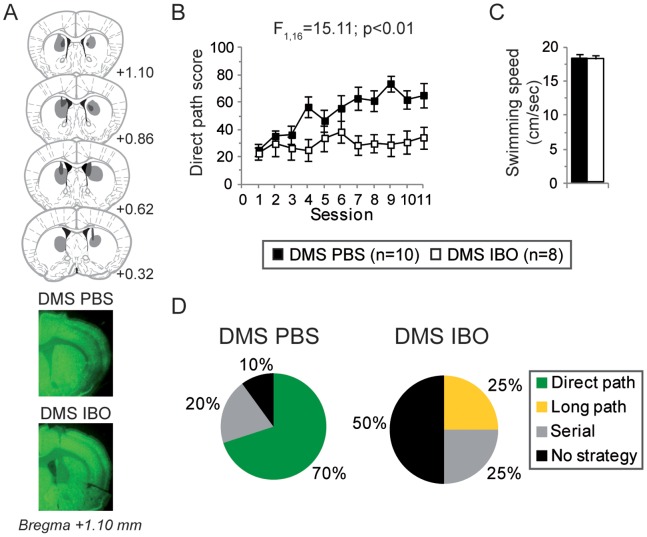
Learning performances of DMS lesioned mice in the starmaze. (**A**) *Upper.* Schematic representation of excitotoxic lesions of the DMS. Shaded areas represent the minimum (dark gray) and the maximum (light gray) extent of the lesions. Ventricles are in black. Numbers indicate distance from Bregma in mm. *Lower.* Representative coronal sections from control (injected with PBS as vehicle, DMS PBS) and dorsomedial striatum-lesioned mice (injected with ibotenic acid, DMS IBO) stained for the neuron-specific marker NeuN (mm anterior to bregma). Infusion of ibotenic acid led to extended neuronal depletion in the dorsomedial part of the striatum. (**B**) Corresponding learning profiles in the starmaze. Compared to PBS-injected mice (closed squares, n = 10), DMS-lesioned mice (open squares, n = 8) exhibited an impaired direct path score. (**C**) There was no difference in swimming speed (black bar: control mice; white bar: DMS lesioned mice). (**D**) Paths most used by the mice during the second half of the training day. PBS mice essentially relied on the direct path (70%) whereas 50% of IBO mice exhibited no clear strategy, 25% used the serial strategy (turning always left at all intersection) and 25% used the long path to the platform (1-2-4-6-7). Only DMS IBO mice displayed the long path.

#### Dorsomedial striatum lesion impairs starmaze task learning

We next examined the effects of DMS lesions in the starmaze task. DMS-lesioned mice displayed neuronal depletion restricted to the dorsomedial part of the striatum with no observable damage to the dorsolateral part (Fig. 4A).

The direct path score of DMS-lesioned mice (n = 8) [DMS IBO] was significantly lower than that of sham control mice (n = 10) [DMS PBS] (two-way ANOVA, *F*_1,16_ = 15.11, *p*<0.01; Fig. 4B) and there was no difference in swimming speed (unpaired t-test, *t*_16_ = -0.04, *p* = 0.95; Fig. 4C). Notably, the direct path score presented no evolution across sessions contrary to hippocampal mice (one-way repeated ANOVA across sessions for control mice, *F*_10,99_ = 4.22, *p*<0.01; for DMS lesioned mice, *F*_10,77_ = 0.32, *p = *0.97; Fig. 4B).

When comparing the paths used during learning by the DMS-lesioned mice and the control mice, DMS mice presented a significant difference of distribution with the control mice (Fisher’s exact test, *p<*0.01). 70% of the control mice used the direct path while no DMS lesioned mice did. Instead, half of them demonstrated no clear strategy (compared to 10% for the PBS mice), a quarter used the serial strategy (compared to 20% for the PBS mice), and the last quarter adopted a different path, the long path to the goal (alleys 1-2-4-6-7) (Fig. 4D).

#### Comparing dorsomedial striatum versus hippocampal lesion

Although both dorsal hippocampus and DMS lesions impaired learning in the starmaze, the behavior exhibited by the lesioned animals was clearly different depending on the lesioned structure, as revealed by the distribution of the paths used (Fig. 3D, 4D). Hippocampal mice were able to develop a serial strategy to reach the goal whereas the DMS mice were lost as shown by the majority of no clear path used. Notably no DMS-lesioned mice learned the direct path to the platform. 25% of them used the non optimal long path to reach the platform. In addition to being non optimal, this path also reveals an inability of the mice to orient themselves towards the platform. Indeed, the configuration of the starmaze is such that when a mouse turns right at the first intersection, it goes towards the opposite direction of the location of the platform (Fig. 1A).

## Discussion

We investigated the structures involved in using the allocentric and sequential egocentric strategies in the starmaze task. Both strategies are encoded in parallel [9], [10] but can be used separately to achieve complex goal-directed navigation in the starmaze task. Regardless of the strategy being adopted, mice were equally rewarded during the training procedure and had to perform the same sequence of body turns to reach the goal. Moreover, mice revealed as allocentric or sequential egocentric learners showed similar learning performances. Therefore, the main difference between using the allocentric and the sequential egocentric strategies resided in what was retrieved by the mice: the association of extra-maze landmarks in the former, and a temporal sequence of body turns in the latter.

Using Fos imaging, we first identified the structures involved in the use of the strategies during the probe test. We then focused on the role of the hippocampus and the DMS in learning the task by lesioning each of these structures.

The profile of Fos expression revealed a common neural network supporting both allocentric and sequential egocentric strategies. This network included the dorsal hippocampus, the DMS and the medial prefrontal cortex (infralimbic and prelimbic areas).

The higher levels of Fos density in infralimbic and prelimbic cortices of mice achieving the starmaze task compared to swimming controls is consistent with the role of these areas in a variety of executive processes such as working memory for spatial location information, decision-making, temporal order memory and planning (see review in [48], [49]) as well as strategy selection [50], [51]. Moreover, the medial prefrontal cortex is part of the associative loop which also includes the DMS and which has been shown to be involved in behavioral flexibility required to solve a goal-directed task [52], [53].

Compared to control mice, Fos activation in the accumbens core was significantly higher only in the sequential egocentric strategy. However a similar tendency was observed in the allocentric strategy, supporting the view that the accumbens subregion is a structure involved in the control of spatial behavior [54], [55], [40].

Interestingly, in the sequential egocentric strategy Fos imaging revealed a specific activation of the ventral CA1 subfield of the hippocampus. As mentioned previously, what distinguishes the sequential egocentric strategy from the allocentric strategy is the recall of a temporal sequence of body turns. Besides the known role of the ventral hippocampus in stress and emotion [56], its differential activation specifically in sequential egocentric mice suggests its functional contribution to temporal order memory as has been shown previously [57].

Elevated Fos expression in the CA1 and CA3 subfields of the dorsal hippocampus of mice using either allocentric or sequential egocentric strategies shows that these areas are involved in the use of either strategies.

Mice with selective dorsal hippocampal lesions were still able to improve their performance in the starmaze task, however they displayed a delayed learning of the direct path to the goal compared to sham controls and never learned the correct sequence to the goal. This deficit was characterized by an inability to learn the correct turn at the second intersection in the starmaze, a turn which is crucial for the temporal organization of the sequence of three body movements (i.e. sequential egocentric strategy) required to ensure successful navigation to the goal. Indeed the hippocampal mice adopted a serial strategy consisting in visiting all radiating alleys successively and systematically reaching the goal. Notice that the serial strategy does not require any learning of the sequential organization of the body movements, an animal using this strategy just explores all encountered alleys by turning toward a defined direction independently of specific choice points. The use of this non-optimal strategy in the starmaze task nevertheless allows reaching the goal doing a small number of errors thus explaining the improvement of the direct path score.

These results, indicative of a navigation deficit affecting both allocentric and sequential egocentric strategies, highlight the role of the dorsal hippocampus in the spatio-temporal organization of information [9]. They are consistent with previous findings showing that the hippocampus is crucial for the acquisition of relationships between multiple stimuli [58], [18] and its well-known role in spatial navigation, namely the representation of the spatial relationships among landmarks which is essential in allocentric strategy [59], [5]. Furthermore these results strengthen the role of the hippocampus in temporal order memory, i.e. for the retrieval of the sequence of self centered body turns required in sequential egocentric strategy [24], [25], [26], [12], see review in [11]. Electrophysiological studies in rodents also reported sequential patterns of firing of hippocampal place cells during spatial navigation [60], [61], [62] as well as firing dependant on the events which occurred earlier or later thus providing temporal information to the spatial representation, such as required in spatio-temporal learning [17], [63].

Our Fos imaging data also revealed the implication of the DMS in the allocentric and sequential egocentric strategies used in the starmaze task. Whatever strategy spontaneously chosen by mice to reach the goal, Fos levels were significantly higher in DMS compared to swimming controls. On the contrary we did not observe any significant DLS activation during the probe test whatever the strategy chosen. To investigate that Fos activation in the DMS highlights its functional involvement in learning the starmaze task, we disrupted the DMS’s function and examined the effects on learning performance in the starmaze. Mice with selective lesions of the DMS were unable to learn the starmaze task. Unlike hippocampal mice, there was no improvement of the direct path score. More specifically, the examination of the paths used during training showed that the majority of DMS mice exhibited no clear strategy. These results bring to light the role of the DMS in learning and using allocentric and sequential egocentric strategies.

The DMS has been shown to play a crucial role in processes mediating goal-directed behavior and decision-making such as action-outcome associations, behavioral flexibility and action selection [27], [64], [65], [66]. Supporting the role of the DMS in goal-directed behaviors, this region has been shown to contain reward-responsive neurons, stimulus-related neurons as well as location-related neurons [67], [68], [69], [70]. In addition, electrophysiological recordings from the dorsomedial striatal neurons in rodents performing a Go/No go reaction time task [71] and caudate neurons in monkeys performing a reaction-time visual motion direction-discrimination task [72] suggested that the caudate nucleus provides necessary signals to evaluate and influence the decision process.

The failure of DMS lesioned mice to learn the starmaze task may be explained by the inability to create a link between a goal localized far from the animal and the spatio-temporal sequence needed to reach that goal. Indeed our data suggest that a functional DMS is required to learn the correct actions leading to the goal, irrespective of the representation used, i.e. spatial or sequential. While most hippocampal mice developed the serial strategy and therefore improved their performances, most DMS mice were unable to adopt any clear strategies. Our result corroborates previous studies arguing that the DMS is engaged in the cognitive control of behavior [28], [73], [74], especially in the selection of action in goal-directed behaviors [8], and plays a role in spatial navigation tasks [29], [30], [31], [35], [68].

Goal-related information has been shown to influence hippocampal place fields [75], [76], [77]. By comparing, in two different tasks, the influence of motivational state (hunger and thirst), memory demand and spatial behavior, Kennedy and Shapiro [78] reported that beyond coding the spatio-temporal context, hippocampal representations can integrate the relationships between internal states, the external environment and actions, thus providing a mechanism coordinating motivation and memory to control goal-directed behavior. This is consistent with findings of Mizumori and colleagues [79], [80] which emphasize that the default mode of hippocampal processing is to continually integrate perceptions of sensory, movement and motivational information within a spatial context.

Early studies on the DMS by Devan et al. [29] led the authors to the conclusion that the DMS was part of a system that includes the hippocampus and may contribute to behavior based on cognitive–spatial forms of information processing. Indeed when given a choice between a visible or an invisible platform, rats with DMS lesions preferentially went to the visible platform, suggesting an inability to learn the position of the invisible platform. At the cellular level, some studies reported in the striatum neurons with firing properties quite comparable to that of hippocampal place cells [68], [81], [82] that are recruited under conditions in which space carries information about the availability of reward [83].

Our data show on the one hand that the goal-related information coding in the hippocampus is not sufficient to allow optimal goal-directed behavior as no mice learned the direct path when their DMS was lesioned, and on the other hand that the DMS does not process spatial information sufficiently to allow learning the direct path to the goal.

Taken together, our results demonstrate that the hippocampus and the DMS are involved in learning a complex goal-directed navigation task: the spatio-temporal organization of information performed by the hippocampus can be processed by the DMS to perform optimal goal-directed navigation and both structures cooperate to perform either allocentric or sequential egocentric strategies successfully.

## Supporting Information

Figure S1 **The control group used to evaluate the nonspecific aspects of the training procedure on expression of the c-fos activity-dependent gene.** Mice were allowed to swim in only one alley, corresponding to the departure alley of the experimental group, namely alley 1 during training trials and alley 5 during probe trials. Swimming occurred in the presence of all visual cues for a duration matching the mean amount of swimming time of the experimental group.
(PDF)

Figure S2 **Significant correlations were found between normalized Fos counts measured in the CA1 (A) or the CA3 (B) fields of the hippocampus and the DMS for all mice groups.** (Black squares: Allocentric mice; Open squares: Sequential egocentric mice; Gray squares: Swimming control mice.)
(PDF)

Figure S3 **Accelerating Rotarod performances.** Performances on the accelerating rotarod were not significantly different for all mice groups. In particular, lesions did not affect motor performances on the rotarod test.
(PDF)

Table S1 **Examples of direct path score calculation for different trajectories.** The direct path to the goal is represented by the sequence of three successive turns following the 1, 10, 8, 7 alleys. A turn score is given for each of the three intersections encountered along the direct path: intersection I (1;10), intersection II (10;8) and intersection III (8;7). The intersection turn score is equal to 100 for a correct turn and 0 for a wrong turn. Each intersection turn score is normalized by the number of alleys visited between the evaluated intersection and the first encounter of the previous one. The normalized turn scores for the three intersections are averaged to obtain the direct path score. This direct path score enables to evaluate the ability of the mouse to navigate directly toward the platform. This actually corresponds to making the correct turn as from the first encountered intersection (intersection I). Not turning to the alley number 10 at the first intersection also reveals an inability of the mouse to orient itself towards the platform. Indeed, the configuration of the starmaze is such that when a mouse turns right at the first intersection, it goes towards the opposite direction from the location of the platform. Thus when considering two serial behaviors, namely left and right serial paths, the former corresponds to a turn towards the platform’s location whereas the later indicates the mouse is swimming away from it.
(PDF)
