# Supplementary figures and images for: Complementary Roles of the Hippocampus and the Dorsomedial Striatum during Spatial and Sequence-Based Navigation Behavior
Source: PLoS One. 2013 Jun 27;8(6):e67232. doi: 10.1371/journal.pone.0067232 (PMC3695082; doi:10.1371/journal.pone.0067232)

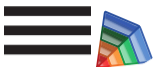

Probe trial

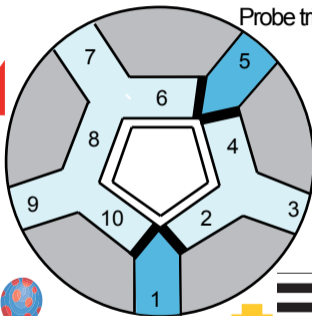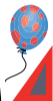

Training

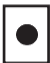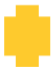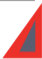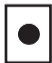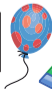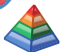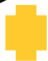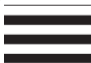

Supplement: Figure S1 — The control group used to evaluate the nonspecific aspects of the training procedure on expression of the c-fos activity-dependent gene. Mice were allowed to swim in only one alley, corresponding to the departure alley of the experimental group, namely alley 1 during training trials and alley 5 during probe trials. Swimming occurred in the presence of all visual cues for a duration matching the mean amount of swimming time of the experimental group. (PDF) [file pone.0067232.s001.pdf]

**A**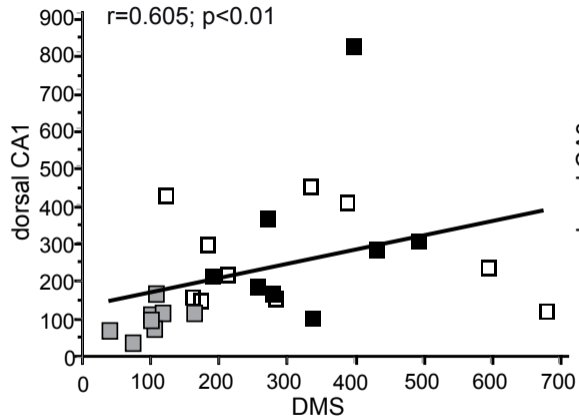**B**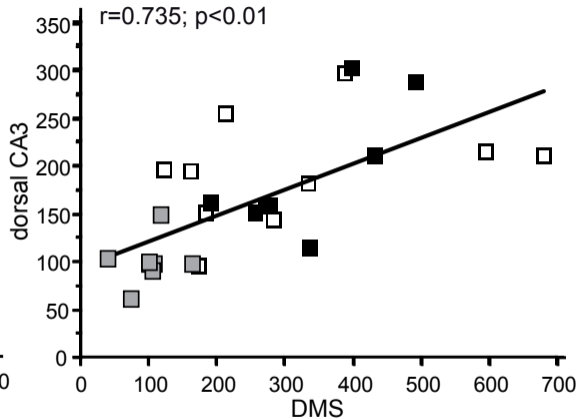

■ Allocentric (n=7)

□ Sequential egocentric (n=9)

■ Swimming controls (n=8)

Supplement: Figure S2 — Significant correlations were found between normalized Fos counts measured in the CA1 (A) or the CA3 (B) fields of the hippocampus and the DMS for all mice groups. (Black squares: Allocentric mice; Open squares: Sequential egocentric mice; Gray squares: Swimming control mice.) (PDF) [file pone.0067232.s002.pdf]

# Accelerating Rotarod

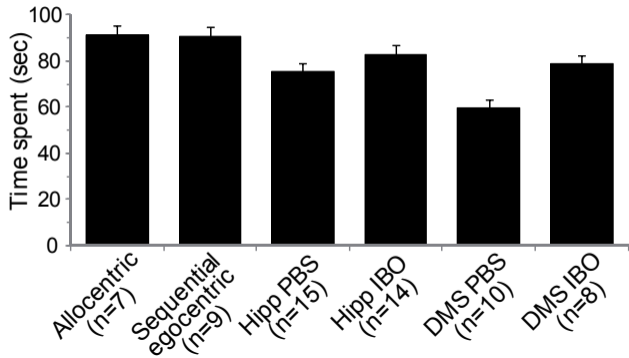

Supplement: Figure S3 — Accelerating Rotarod performances. Performances on the accelerating rotarod were not significantly different for all mice groups. In particular, lesions did not affect motor performances on the rotarod test. (PDF) [file pone.0067232.s003.pdf]
