# Supplementary material for: Complementary Roles of the Hippocampus and the Dorsomedial Striatum during Spatial and Sequence-Based Navigation Behavior
Source: PLoS One. 2013 Jun 27;8(6):e67232. doi: 10.1371/journal.pone.0067232 (PMC3695082; doi:10.1371/journal.pone.0067232)
Supplement: Table S1 — Examples of direct path score calculation for different trajectories. The direct path to the goal is represented by the sequence of three successive turns following the 1, 10, 8, 7 alleys. A turn score is given for each of the three intersections encountered along the direct path: intersection I (1;10), intersection II (10;8) and intersection III (8;7). The intersection turn score is equal to 100 for a correct turn and 0 for a wrong turn. Each intersection turn score is normalized by the number of alleys visited between the evaluated intersection and the first encounter of the previous one. The normalized turn scores for the three intersections are averaged to obtain the direct path score. This direct path score enables to evaluate the ability of the mouse to navigate directly toward the platform. This actually corresponds to making the correct turn as from the first encountered intersection (intersection I). Not turning to the alley number 10 at the first intersection also reveals an inability of the mouse to orient itself towards the platform. Indeed, the configuration of the starmaze is such that when a mouse turns right at the first intersection, it goes towards the opposite direction from the location of the platform. Thus when considering two serial behaviors, namely left and right serial paths, the former corresponds to a turn towards the platform’s location whereas the later indicates the mouse is swimming away from it. (PDF) [file pone.0067232.s004.pdf]

Path	Intersection I turn score	Intersection II turn score	Intersection III turn score	Direct path score
**1-10-8-7 (Direct path)**	100/1	100/1	100/1	**100**
**1-10-8-6-7**	100/1	100/1	0	**66.7**
**1-10-9-8-7 (Left serial path)**	100/1	0	100/2	**50**
**1-2-10-9-8-7**	0	0	100/2	**16.7**
**1-2-4-6-7 (Long path)**	0	-	-	**0**
**1-2-3-4-5-6-7 (Right serial path)**	0	-	-	**0**
